# Editorial: Allorecognition by Leukocytes of the Adaptive Immune System

**DOI:** 10.3389/fimmu.2017.01555

**Published:** 2017-11-15

**Authors:** Gilles Benichou, James Kim

**Affiliations:** ^1^Center for Transplantation Sciences, Department of Surgery, Massachusetts General Hospital, Harvard Medical School, Boston, MA, United States

**Keywords:** allorecognition, lymphocytes, transplantation immunology, tolerance, MHC

**Editorial on the Research Topic**

**Allorecognition by Leukocytes of the Adaptive Immune System**

Allorecognition refers to a series of mechanisms by which an individual’s immune system distinguishes its own cells and tissue from those of another individual belonging to the same species. This phenomenon is responsible for self–non-self recognition in both invertebrates and vertebrates. Both cells of innate and adaptive immune systems are capable of allorecognition.

In vertebrates, allogeneic cells are recognized by lymphocytes of the adaptive immune system, through interaction of alloantigens (antigens expressed by allogeneic but not autologous cells) with specific receptors (BCR and TCR for B and T lymphocytes, respectively). Such allorecognition by lymphocytes can trigger immune responses, which can lead to either rejection or acceptance of cells, tissues, and organs displaying these alloantigens. In the case of transplantation of allogeneic organs and tissues, allorecognition generally initiates an inflammatory response, which leads to the destruction and rejection of the graft. On the other hand, a number of regulatory mechanisms have been selected through evolution to suppress deleterious alloimmunity in selected situations. For instance, during pregnancy in mammalians, alloimmunity directed to paternal antigens generally prevents immune attack and abortion of the fetus by the female’s immune system. Similar to the fetus, it is now established that allorecognition within certain organs such as the testis and the brain (called immune privileged) triggers specific types of immune responses protecting allogeneic cells from rejection. This phenomenon called immunological tolerance involves active processes mediated by regulatory lymphocytes and cytokines. Therefore, the nature of the cells, the environment, and the molecular mechanisms involved in allorecognition govern the fate of the immune response to allogeneic cells (rejection or tolerance).

This Topic of Frontiers offers the reader views on key aspects of the immune mechanisms underlying allorecognition by T and B cells and its relationship to the development of pro-inflammatory and regulatory responses involved in rejection or tolerance of allografts.

T cells are considered as the main driving force behind the initiation and regulation of immunity to alloantigens. The article by Jose Marino and his colleagues reviews current knowledge regarding the different pathways of alloantigen presentation, direct, indirect, and semi-direct, and their contributions to T cell alloimmunity and allograft rejection. This paper provides evidence that the role of different allorecognition pathways in allograft rejection or tolerance varies depending upon the nature of the transplant, its site of placement, and the time after transplantation. This paper also describes recent studies describing how extracellular vesicles and donor MHC cross-dressing of recipient cells influence allorecognition and allograft rejection. The paper by Gilles Benichou et al. summarizes current knowledge regarding alloresponses by memory T cells in experimental and clinical transplantation models. The article by Sehrawat and Rouse describes the interplay of regulatory T cells and CD4^+^ TH17 cells and how it influences the fate of immune responses in humans and animals. The article by Degauque and his colleagues describes different studies designed to track and identify particular T cell clones involved in allograft rejection or tolerance. Finally, the paper by Scalea et al. summarizes current knowledge of cell therapies designed to achieve T cell tolerance in transplantation and their mechanisms of action.

The concept of immune privilege is an important aspect of transplantation immunology. Indeed, certain organ and tissue transplants are less susceptible than others to inflammation and rejection. At the same time, placement of allografts in selected body sites enjoy long-term survival with no or minimal immunosuppression. Immune privilege represents a natural form of tolerance selected through evolution to prevent potentially dangerous inflammation in selected tissues and organs, such as the central nervous system. It is also an important element of the prevention of immune attack of the fetus and its abortion by the mother during pregnancy. Therefore, a better understanding of the mechanisms underlying this phenomenon may help with the design of tolerance protocols in transplantation. The paper by A. Taylor summarizes current knowledge in the field of immune privilege and addresses specific mechanisms contributing to the development and maintenance of immune privilege in the eye.

B lymphocytes play a key role in the response to and rejection of allogeneic transplants. They contribute to this process by producing antibodies against donor MHC antigens and tissue-specific autoantibodies and by serving as antigen-presenting cells for T cell activation. In addition, there is accumulating evidence showing that donor-specific antibodies and autoantibodies are involved in chronic form of allograft rejection characterized by tissue graft fibrosis and vasculopathy, a major cause of progressive organ transplant failure in clinical settings. On the other hand, recent studies have now firmly established the existence of anti-inflammatory, tolerogenic B cells (regulatory B cells or Bregs), which contribute, along with regulatory T cells, to prevent allograft rejection. The article by Michelle Hickey and colleagues provides a comprehensive overview on the generation and functions of alloantibodies directed to HLA and their role in transplant rejection. The paper by Daniel Firl et al. discusses current knowledge of different subsets of B cells, including regulatory B cells, in rejection and tolerance of allografts in experimental and clinical transplantation models.

Antigen-presenting cells, in particular dendritic cells (DCs), initiate alloimmune responses by activating T cells through the presentation of intact donor MHC molecules (direct allorecognition) or donor MHC peptides (indirect allorecognition). It is now well established that the nature of the T cell responses depends on the nature of the DCs, their degree of maturation, and their ability to deliver selected costimulatory signals to T cells. The article by Angus Thomson and colleagues reviews current knowledge regarding the tolerogenic properties of certain DCs and their potential utilization to achieve tolerance in transplantation.

The transplantation of allogeneic bone marrow and stem cells is an important aspect of clinical transplantation as it has been used for curative treatment of hematological malignancies. Unfortunately, the desired antitumor or graft-versus-leukemia effect is often accompanied with undesired side effects against healthy tissues known as graft-versus-host disease. The article by M. Griffioen and colleagues provides insights into the composition and kinetics of *in vivo* immune responses with respect to specificity, diversity, and frequency of specific T-cells and surface expression of HLA–peptide complexes and other (accessory) molecules on the target cell. It describes how the complex interplay between these factors and their environment ultimately determines the spectrum of clinical manifestations caused by immune responses after transplantation of allogeneic stem cells.

In summary, great progress has been made with regards to the description of the cells and molecules involved in allorecognition by the adaptive immune system. Furthermore, we have acquired a better understanding of the mechanisms by which these cells and molecules influence the nature of the immune response to alloantigens in health and disease. Based on this knowledge, novel strategies are being designed in an effort to manipulate alloreactivity in clinical settings, such as organ transplantation and transplantation of allogeneic bone marrow and stem cells as well as pancreatic islet cells.

## Author Contributions

All authors listed have made a substantial, direct, and intellectual contribution to the work and approved it for publication.

## Conflict of Interest Statement

The authors declare that the research was conducted in the absence of any commercial or financial relationships that could be construed as a potential conflict of interest.

